# Continuous Glucose Monitoring for the Diagnosis of Gestational Diabetes Mellitus: A Pilot Study

**DOI:** 10.1155/2022/5142918

**Published:** 2022-10-17

**Authors:** Daria Di Filippo, Marrwah Ahmadzai, Melissa Han Yiin Chang, Ksana Horgan, Ru Min Ong, Justine Darling, Mahmood Akhtar, Amanda Henry, Alec Welsh

**Affiliations:** ^1^School of Women's and Children's Health, University of New South Wales Sydney, Locked Bag 2000, Barker Street, Randwick, NSW 2031, Australia; ^2^Diabetes Clinic, Royal Hospital for Women, Barker street-Randwick, NSW 2031, Australia; ^3^Department of Maternal-Fetal Medicine, Royal Hospital for Women, Barker street-Randwick, NSW 2031, Australia

## Abstract

**Background:**

Gestational diabetes mellitus (GDM) is diabetes first diagnosed in pregnancy. GDM, together with its short- and long-term negative outcomes, is increasing in incidence all over the world. The current diagnostic method for GDM, the oral glucose tolerance test (OGTT), is dated and has been reported as inconvenient for women as well as poorly reproducible and reliable.

**Aims:**

We aimed at assessing the acceptability, feasibility, and accuracy of continuous glucose monitoring (CGM) as a diagnostic test for GDM and explore its correlation with the OGTT and risk factors for GDM.

**Methods:**

In this prospective cohort study, pregnant women due for or having completed OGTT underwent CGM for seven days, performing daily finger-prick blood glucose levels before completing an acceptability questionnaire. Data on GDM risk factors and CGM variability were analyzed and compared with OGTT results.

**Results:**

Seventy-three women completed CGM (40 GDM, 33 normal glucose tolerances); 34 concurrently underwent OGTT. CGM was acceptable and generally well-tolerated, with skin irritation/itchiness the only adverse event (11 mild, one severe). CGM and OGTT strongly correlated for fasting glucose values (*r* = 0.86, *p* < 0.05) only. Triangulating GDM risk factors, OGTT results and CGM variability parameters with the application of machine learning highlighted the possibility of unmasking false positive (11 showed low CGM variability and demographic risks but positive OGTT) and false-negative OGTT diagnoses (1 showed high CGM variability and demographic risks but negative OGTT).

**Conclusions:**

CGM was well-tolerated, showing poorer glycaemic control in GDM, and revealing potential misdiagnosis of the OGTT when combined with GDM risk factors. Future research is needed to determine cut-off values for CGM-defined and OGTT-independent screening criteria for GDM.

## 1. Introduction

Gestational diabetes mellitus (GDM) is glucose intolerance first recognized during pregnancy that typically resolves thereafter [1]. It reflects maternal physiological inability to compensate for the progressive insulin resistance of pregnancy [2]. GDM is the fastest-growing type of diabetes in Australia and affects one in six pregnancies globally [3].

A significant, continuous correlation exists between maternal hyperglycemia and adverse perinatal outcomes [4]. GDM means increased monitoring and often interventions for mothers, including labor induction or caesarean section, and increased risk of obstetric complications (perineal lacerations and preeclampsia) [5].

The anabolic effects of maternal-fetal hyperglycemia and hyperinsulinemia increase the risk of macrosomia (birthweight >4 kgs), large for gestational age (birth weight > 90th centile) and obstructive delivery complications [4]. Additional neonatal risks are induced prematurity, newborn respiratory distress syndrome and metabolic disorders (hypoglycaemia, hyperinsulinemia and hyperbilirubinemia) [4, 6]. Mother and newborn are susceptible to later Type 2 Diabetes [7].

Within Australia, pregnant women undergo the oral glucose tolerance test (OGTT) at 24-28 weeks gestation or, if at high-risk, at 12-16 weeks and again at 24-28 weeks, if initially negative [8]. OGTT reliability has been questioned, as it involves a supraphysiological load unrelated to body weight or normal/necessary dietary intake [9]. Being unpleasant, expensive, and time-consuming, OGTT has poor reproducibility: 25% of women with positive results have negative results when re-tested [9, 10]. False positive women, maintaining normal blood glucose level (BGL) on their usual diet, risk overtreatment, with increased stress, healthcare workload, and costs [11]. Of greater concern are potential false-negative diagnoses in women with habitually high carbohydrate diets, undetected by the seemingly low sugar load of their OGTT and lacking treatment/prevention of GDM complications.

Continuous glucose monitoring (CGM) involves a disposable subcutaneous electrochemical sensor measuring interstitial glucose based on its reaction to glucose oxidase. The sensor is connected to a receiver to measure and store interstitial glucose data automatically [12]. Average values can be displayed on a monitor at ~5-minute intervals, yielding ~288 measurements daily for the Medtronic iPro2 and Dexcom G5, or every 15 minutes for the FreeStyle Libre Pro [13–15]. Resulting data is accessible as an Excel list of measurements or a continuous curve, mapping magnitude, frequency, and duration of glucose excursions [16].

CGM has been used in gestational diabetic women primarily as a management tool allowing a more acceptable and reliable glucose reading and control than self-monitoring of blood glucose (SMBG), as reported in a recent systematic review [17].

With a view to progress the role for CGM as a diagnostic tool for GDM, the primary objective of this pilot study was to formally assess the acceptability and tolerability of Medtronic iPro 2 CGM among a general pregnant population, including, but not limited to, patients diagnosed with GDM. Secondary aims included evaluating differences in glycaemic variability among GDM and normal glucose tolerance (NGT) patients and triangulating risk factors, OGTT and CGM results by applying unique machine learning techniques.

## 2. Materials and Methods

### 2.1. Research Design

This prospective cohort study was conducted at a tertiary metropolitan Sydney hospital between February 2015 and October 2017. Women between 12- and 35-weeks' gestation were recruited through the antenatal clinic at GDM education groups or through midwifery referral according to the following inclusion criteria are as follows: women recently diagnosed with GDM at their routine OGTT, both before and after their first GDM education, or those willing to undergo OGTT during CGM monitoring. The OGTT was performed using a 75 grams glucose beverage. Women were subsequently divided into two groups, GDM or NGT, based on their OGTT results interpreted using the International Association of the Diabetes and Pregnancy Study Groups (IADPSG) criteria [18]. Exclusion criteria were preexisting Type 1 or Type 2 diabetes, mental illness precluding informed consent and pharmacological management of GDM (insulin or metformin) at time of CGM monitoring (women in the first month after GDM diagnosis who had received some nonpharmacological management only were eligible). All women provided written consent to participate after receiving further information in an initial consultation.

### 2.2. Research Equipment and Process

CGM was performed with the Medtronic iPro2 system (Medtronic, Northbridge, CA) [19]. Following iPro2 insertion on the abdomen, participants received instruction on performing device calibration, and SMBG with finger-prick glucose measurement at least twice daily. Participants were provided with daily logs for SMBG and instructed to record the timing of meals, exercise, and medication in a paper diary. The monitoring period consisted of 7 days, following which participants returned to the antenatal clinic to remove the monitor and complete a questionnaire regarding device acceptability (Supplementary material 1). The (nonvalidated) bespoke survey consisted of five questions regarding the overall acceptability as well as acceptability of insertion, wearing and removal and the likelihood of recommending CGM as a diagnostic test for other women in a Likert scale format of 0-5. A final free text box allowed participants to share any recommendation or comment.

### 2.3. Data Collection and Analysis

Clinical data was obtained from the hospital's obstetric database (Supplementary material 2). Data from the iPro 2 was uploaded to web-based software (CareLink iPro, Medtronic) and exported for analysis [20]. Statistical analysis was performed using Microsoft Excel (Microsoft, WA, USA), SPSS (SPSS Inc., IL, USA), and MATLAB (version R2019a, MathWorks Inc., Natick, MA, USA) [21, 22]. Glycaemic reports generated for each patient in Microsoft Excel were individually considered to determine validity for analysis. CGM data were excluded if the 24 hour CGM and SMBG mean differed by more than 28%, as suggested by the accuracy data reported by the CGM manufacturer [23]. Of the seven complete continuous days of CGM, only the first three consecutive days (excluding the day of the OGTT), containing the full 288 CGM measurements per day, were considered valid and analyzed. The CGM parameters considered in our analysis are outlined in Table 1. Day time values were considered from 06: 00 to 23 : 59 hrs and night-time values from 00 : 00 to 05 : 59 hrs. The follow up ended at birth. To be included in final data analysis, OGTT date and results must have been available.

### 2.4. Bias

CGM data was analyzed for a period of three days only to exclude the day of the OGTT, which would have given nonreliable readings, as suggested by our OGTT/CGM correlation analysis and to evaluate the highest number of women possible in our cohort.

### 2.5. Power Calculation

As data generated by this study were pilot in nature to power future studies, conducting a power calculation was complex and somewhat hypothetical. We estimated a sample size using G-Power (University of Dusseldorf, 2007) based upon the association between the three BGL assessed with OGTT (0, 1, 2 hours) and the corresponding values at CGM [26]. This indicated that a sample size of 26 women undergoing both OGTT and CGM would be sufficient to detect a large (effect size 0.5) difference in the correlation of OGTT and CGM results, with a power of 80% and an alpha significance level of 0.05.

Normally distributed continuous variables are presented as mean ± standard deviation (SD); nonnormally distributed continuous variables are presented as median with interquartile range. Continuous variables were compared between groups using *t*-test (normally distributed) and Mann–Whitney *U* test (nonnormally distributed) as appropriate. Categorical variables are presented as percentages and were compared using Chi-Square or Fisher's exact test as appropriate. Values of *p* < 0.05 were considered statistically significant. Correlation between paired fasting, 1 hour and 2 hour, OGTT and CGM values was performed using Pearson's R correlation in a subgroup of 13 NGT patients, for whom the precise OGTT timing was available. For the OGTT-CGM values correlation, we considered the difference between the OGTT value and the mean of a 20-minute period represented by the 2 values before and 2 after the exact timing of the OGTT blood drawn. This was an empirical attempt to accommodate for CGM shifts of BGL measurement.

### 2.6. Machine Learning Analysis

Supervised machine learning for our automatic classification process was used with various classifiers in MATLAB [22]. A combination of representative features for each participant was selected (i.e., defining a multidimensional feature space) to train models and validate them using 5-fold cross-validation (i.e., partitioning the data set into 5-folds and estimating accuracy on each fold while training the model on remaining 4-folds) to protect models against overfitting. Four novel risk parameters were computed for each participant based on patient demographics such as ethnicity, age, weight, family GDM history, and personal GDM history. Finally, a combined demographic risk factors score (CDRFS) was computed by applying their correlation with GDM diagnosis from existing literature. With an assumption that the baseline risk = 1, the individual novel risk parameters were derived through the following rationale:

- For each one-year increase in maternal age from 18 years, the GDM risk for the overall population, Asian population and European population increases, respectively, by 7.9%, 12.74%, and 6.52% [27]
- If a person is overweight or obese and older than 35 years, the GDM risk is 2.45 times higher than baseline [28]
- If a person has a family history of diabetes mellitus and is more than 30 years old, the GDM risk is three times higher than baseline [29]
- If a person has previous GDM history, the risk is 5.24 times higher than baseline [30]

The classification learner application in MATLAB [22] was used to train multiple machine learning models (support vector machines (SVMs), K-nearest neighbors (KNN) algorithms, ensemble algorithms) to automatically classify GDM positive and negative participants while using OGTT results as the underlying ground truth. After exploring 4 different options of CGM parameters combination, we also calculated CGM scores of variability (CGMSV 1-4). For CGMSV and CDRFS cut-offs, we determined the midpoint between the maximum of the NGT group and the minimum of the GDM group on the normalized values. We then triangulated the OGTT results with both CDRFS and CGMSV 1-4 in the 60 women for who complete information on risk factors were available.

## 3. Results and Discussion

### 3.1. Cohort Composition and Demographics

**Figure 1:**
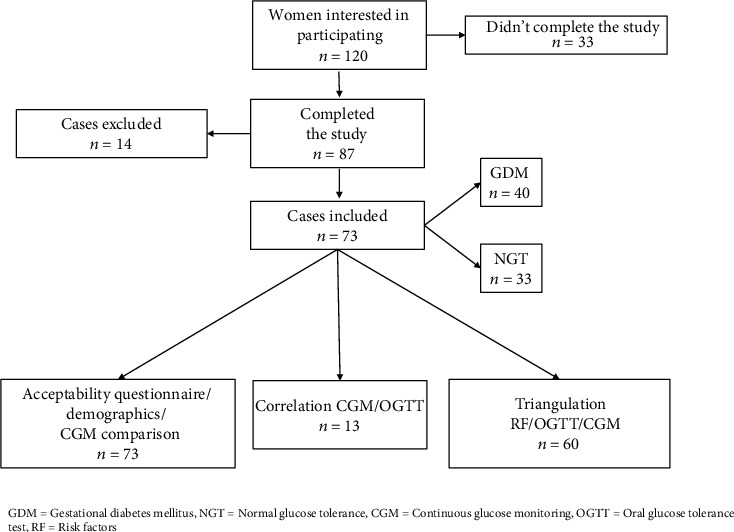
Consort diagram.

Of a total of 120 women interested in participating, 73 (61%) women completed the study and had their data included for analysis as follows: 40 GDM and 33 NGT (Figure 1).

Of the 47 women excluded, 33 did not complete the study due to social reasons including transport difficulties, young children, hectic work schedule, unwillingness to commit to extra appointments, or keeping a logbook. Fourteen were excluded from analysis due to insufficient days of CGM (*n* = 5), missing finger prick calibrations (*n* = 3), missing OGTT details (*n* = 5), and one intrauterine fetal death. Demographic data and CGM variability were compared in the final 73 inclusions. Demographic data are summarized in Table 2.

There were more primigravida in the NGT group (78% vs 42% in GDM group, *p* < 0.01), and a significantly higher proportion of GDM participants had a family history of diabetes compared to NGT (57% vs 23%, *p* < 0.01). No difference in terms of birthweight was found between the two groups.

### 3.2. CGM Acceptability

CGM was well-tolerated as follows: 75% of participants gave a rating of 5/5 for overall device acceptability, 83% for device insertion, and 85% for removal (Supplementary material 1). Only 63% rated 5/5 for the duration of wearing the device though 82% would definitely recommend CGM testing for GDM to other pregnant women. Within the free text section of the questionnaire, the majority of the participants reported that the device was barely noticeable. Eleven women (15%) mentioned mild irritation and itchiness due to device and/or dressing during the last 1-2 days of the monitoring period, and one patient reported severe itchiness. Only one woman stated that she preferred the OGTT over CGM.

### 3.3. CGM and OGTT Results

The amount of CGM data (as CGM wearing days and glucose values) collected was higher for GDM than NGT women (5.3 vs 4.8 days and 1526 vs 1318 values, respectively, both *p* < 0.05). Only nineteen of the seventy-three women included, all classified as having GDM, performed 4 measurements per day. There was no statistically significant difference of the delta between SBGM and sensor measurements of glucose values between women with at least and less than 4 measurements per day (9.9% + /−4.6 vs 8.8% + /−4.3, *p* = 0.385). The first night of CGM monitoring following insertion was evaluated for forty-six women (of which 38 = 83% already diagnosed with GDM), of whom 15 experienced some degree of hypoglycaemia during the first night only. Among the twenty-seven women for which the first night of CGM monitoring following insertion was not evaluated, 4 had hypoglycaemia only during night one. This difference was found to be not statistically significant (32.6.4% vs 14.8%, *p* = 0.079). There was no significant difference of night one mean sensor BGL between the 46 women for which the first night of monitoring was included versus the 27 for which it was not included (4.57 vs 4.86, *p* = 0.288). The mean sensor BGL of night 1 was found to be significantly lower than night 2 and 3 for women in which the first night of CGM monitoring was included (4.57 + /−1.35 vs 4.91 + /−0.99 vs 5.00 + /−0.95, *p* < 0.001). Conversely, night one mean sensor BGL was significantly higher in women for which the first night of CGM monitoring following insertion was not included (4.86 + /−0.95 vs 4.81 + /−0.60, *p* < 0.001). The mean delta between SBGM and CGM measurements for the three days and nights included, as well as for the first measurement taken in the morning of day 2, was lower in women for whom night one corresponded with the first night of CGM wearing (8.56 + /−3.93 vs 9.76 + /−4.39 and 8.91 + /−6.36 vs 10.84 + /−7.94), although not statistically significant.

Measures of glucose and glycaemic variability are shown in Table 3.

All were higher in the GDM than the NGT group: mean glucose value 5.53 mmol/L ± 0.9 SD vs 5.06 mmol/L ± 0.8 SD, *p* = 0.03; MODD 0.96 vs 0.75 (*p* < 0.01); MAGE 2.33 vs 1.73 (*p* < 0.01). The GDM group had more women having time below and above (<3.5 and> 7.8 mmol/L) the range both during the day and during the night, although this only reached statistical significance for TAR (67% vs 30%, *p* = 0.002) and women with>70% of TIR (72% vs 100%, *p* = 0.001) during the day (Table 4).

GDM women spent more time below and above the range than NGT women, with lower time spent in range in total and during the day although none of these results were statistically significant (Supplementary material 3).

#### 3.3.1. CGM and OGTT Results: Correlation

A strong positive correlation (*r* = 0.86, *p* < 0.05) was seen between fasting OGTT and CGM value after removal of a single outlier. Correlation between CGM and OGTT weakened and was nonsignificant at the 1 hr and 2 hr postglucose load mark (*r* = 0.38 and 0.47, respectively).

#### 3.3.2. CGM and OGTT Results: Prediction

Models were trained and tested on a multidimensional dataset of 60 patients (27 NGT and 33 GDM) for whom the risk factors data were fully available [29–31]. The combination of CGM parameters of variability (sensor mean, SD of the mean, and MAGE) gave up to 82% accurate prediction of GDM positive (true positive rate) and up to 77% accurate prediction of GDM negative (true negative rate) participants, using ensemble machine learning RUSBoost algorithms [32] with a false-positive rate of 18% and false-negative rate of 23% (Supplementary material 4).

### 3.4. CGM, OGTT and Risk Factors

#### 3.4.1. Combined Demographic Risk Factor Score

The MATLAB code used to generate demographic risk factors scores and a CDRFS based on the correlation between single risk factors and GDM in literature can be found as a supplementary figure (Supplementary material 5). Linear regression showed significant correlations (*p* < 0.05) between each of these scores and GDM: CDRFS the highest (*R*^2^ = 0.097), followed by family history/age (*R*^2^ = 0.078), and age/ethnicity (*R*^2^ = 0.073) (Supplementary material 6).

### 3.5. CGM Score of Variability

Four different CGMSV were evaluated. These were based on as follows:

1. correlation of CGM parameters with OGTT results
2. the ranges for the general population
3. the sum of the normalized values of the CGM variability parameters (sensor mean, SD, CV, MODD, MAGE)
4. the normalized percentages of time (expressed in decimals) spent in/below/above the range recommended for pregnancy in total and during the day (06 : 00 to 23 : 59) and the night (00 : 00 to 05 : 59) (Supplementary material 7) [33]

**Figure 2:**
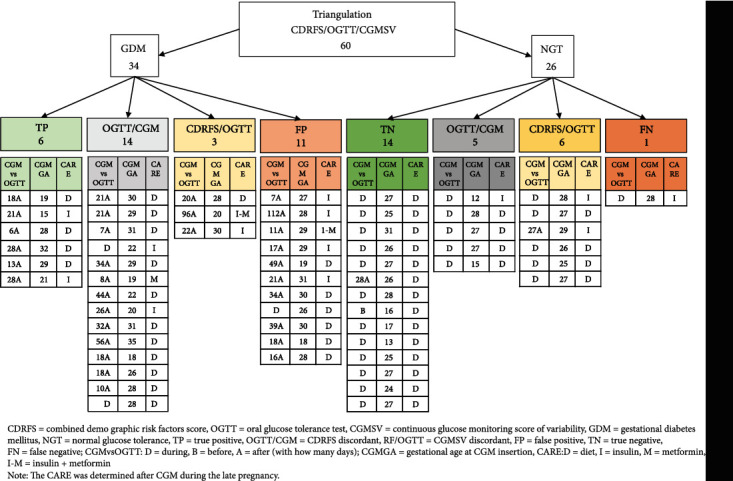
Results of CDRFS, OGTT results, and CGMSV triangulation.

The correlation with OGTT was higher for CGMSV1 than CGMSV4 (Supplementary material 8). CGMSV4 was chosen for the final triangulation (as presented in Figure 2), being based on variability parameters of CGM and ranges recommended for pregnant women independent from their ability to predict OGTT. Results of the correlation with all the CGM scores of variability can be found in Supplementary material 9. This manuscript was prepared in accordance with the STROBE checklist (Supplementary material 10) [34].

### 3.6. CGM, OGTT and Risk Factors Triangulation

Figure 2 shows the comparison between OGTT results and both demographic and CGM variability risk score 4 in 60 women, together with data of the additional considerations described below.

Of the 34 women with positive OGTT, only 6 indicated certain true positivity with both CRDFS and CGMSV above the cut-off. Eleven of the women diagnosed as GDM by OGTT had instead low CDRFS and CGMSV indicating a potential misdiagnosis (false positive). Among the 26 NGT women, 14 had concordant demographic and CGM scores below the cut-off, indicating a likely true negative diagnosis, whereas one had high CDRFS and CGMSV suggesting a false negative diagnosis.

### 3.7. Additional Considerations

#### 3.7.1. CGM Monitoring Timing

Most of the GDM women for whom the triangulation was completed (31/34) underwent CGM after their OGTT, on average 26 days later at about 28 weeks. Only 2 of the 26 NGT patients underwent CGM after their OGTT (average 27 days later) at about 26 weeks. The remaining patients underwent CGM during or before their OGTT.

#### 3.7.2. Management

Fifteen women of the triangulation analysis were treated with insulin, three in combination with metformin. Four of these women were among the 26 NGT women included in the triangulation; these women were treated clinically as having GDM after a negative OGTT. None of the 14 women considered true negative required insulin, compared to one of the five women with low CGMSV (who wore CGM at 12 weeks), two of the six women with low CDRFS and negative initial OGTT (but CGM suggesting GDM), and the single woman labelled as false negative. Insulin was used also in five of eleven women considered false positive at screening (low risk and CGM scores but positive OGTT), and two of the three women with high-risk score, positive OGTT but CGM showing good variability and time in range, (potential false positive) as shown in Supplementary material 6.

#### 3.7.3. Main Results

In our cohort of pregnant women, CGM was shown to be an acceptable and feasible alternative to OGTT for GDM diagnosis, fulfilling the primary objective of this study. In addition, CGM also revealed statistically significant differences between GDM and NGT women in terms of BGL variability and time spent in, below, and above the recommended range for pregnant women [33]. Our novel use of machine learning additionally revealed the potential for CGM to identify OGTT false positive and negative diagnosis in a subgroup of 60 patients, although this requires further investigation and refinement.

#### 3.7.4. CGM Acceptability

A previous study evaluating CGM as an educational tool for GDM women reported that 90% of included women accepted the device, with the main limitations being technical difficulties and discomfort with the sensor (worn on the upper buttocks in that case) [35]. A systematic review on the application and utility of CGM in pregnancy as a management tool for GDM also reported good acceptability in two studies [17]. With the aim at assessing CGM as a diagnostic tool for GDM, it was essential for us to evaluate its acceptability for the general pregnant population and not only among GDM women. In the absence of a prespecified optimal duration for CGM detection of GDM, we chose duration of CGM of a maximum of 7 days, and not 14 as per its original use in diabetic people, to maximize CGM acceptability.

CGM insertion and removal were extremely well-tolerated in our study group, the overall period of wearing the device somewhat less so. Approximately one in 6 participants had minor skin irritation, predominantly in the last 1-2 days of wearing. New generation CGM devices wearable in less sensitive areas than the pregnant abdomen and not requiring finger prick calibration may further improve acceptability for patients. The fact that GDM women had a longer CGM wearing time might reflect how women at risk and diagnosed with GDM were keener to wear the sensor for a longer period as more aware of the advantages related to it. The length of CGM wearing was not correlated instead with the parity.

#### 3.7.5. OGTT Acceptability and Completion Rates

CGM could be better tolerated than OGTT, given its limitation for both patients and clinicians. A 2019 Western Australian retrospective study on OGTT completion rates demonstrated that only 50.5% of the files examined showed full completion of the OGTT [24]; reasons included women not liking the idea of a large sugar load, nausea and vomiting, and logistical difficulties (lack of transport, family demands, no time). Measures proposed included offering alternative tests (GCT or HbA1c) as a compromise or routine use of random BSL measurements [24]. Other studies reported a 33-94% screening completion rate in Canada, Israel, and the USA [25, 36, 37]. The Canadian article [25] did not report any reason precluding completion of the screening in almost 20% of patients, as the American one [37], for which the screening completion was reported to be 68%, focused on the maternal characteristics linked to a higher chance to be screened: ages 35-40, weight 100-124 pounds, and Asian ethnicity.

#### 3.7.6. CGM Variability Parameters

CGM patterns reflect the abnormal glycaemia seen in GDM patients versus NGT. Several CGM measures have been documented in the literature [38–40]; we included only the most commonly used (mean, SD, CV, MAGE, MODD and TAR, TBR) to reduce the risk of type 1 statistical error. In a recent study, mean glucose level at CGM was found to be correlated with having large for gestational age newborns [41]. In our cohort, GDM patients not only had higher glucose levels overall than the NGT women but also greater levels of variability within and between different days of monitoring, as indicated by MAGE and MODD, respectively. These results are comparable to a study by Yu et al. [42] exploring the association between CGM variability and pregnancy outcome, [42].

#### 3.7.7. CGM Ranges

Glycaemic targets for GDM women are stricter than those for type one diabetic patients and the general population, although little evidence has been reported so far on GDM tailored thresholds [33]. We found GDM women to spend more time above and below the range and to be less likely in range for at least 70% of the time, compared to NGT women. These results are in line with the output of a recent study by Singh focused on the description of CGM parameters in GDM vs NGT women between 8-20 weeks, which described GDM women to be less likely within the recommended range (92.1% vs 98.2% of the time) [43]. The introduction of GDM tailored ranges in our CGMSV gave us discordant results in terms of prediction among our proposed false positives and negatives. Worthy of note is the fact that the current ranges recommended by ADA to determine the use of insulin are based on a study of 1995 comparing the use of insulin to control premeals or postmeals BGL [44, 45]. The analysis of time in, below, and above range is performed over 24 hours. The analysis of time in range expanded our triangulation and could help in overcoming the OGTT classification. Further studies on the association of CGM ranges, GDM biomarkers and outcomes different from insulin use are needed.

#### 3.7.8. CGM and OGTT Correlation

The correlation between OGTT and CGM mean sensor values explored in a subgroup of 13 NGT women, after removing the outliers, was significantly strong (0.86, *p* < 0.05) only between OGTT time 0 (before the glucose load) and CGM values. This supports CGM being a true reflection of women's glucose values in standard conditions. In contrast, this correlation is lost after a supra-physiological load of sugar in excess of usual dietary intake.

#### 3.7.9. CGM as a Diagnostic Tool

To our knowledge, only three prior studies have evaluated the use of CGM as a diagnostic tool for GDM, comparing it to the OGTT. The first study, involving only nine patients, concluded that CGM was highly tolerated and showed dysglycaemia on 2 of 9 OGTT negative patients [26]. A study of 28 women (20 GDM, 8 controls) reported that glucose fluctuations at CGM for women positive to OGTT once at home did not differ from those having a negative OGTT result, potentially revealing false positives of the OGTT [46].

A more recent study from Tartaglione et al. applied CGM (Medtronic iPro2) to the analysis of postprandial and fasting BGL in a cohort of 99 women seven days after the OGTT [47]. Among the 53 NGT, 33 women were found to have BGL above or below the recommended thresholds and were managed with a one week of SMBG and diet. Twelve of these women ended up requiring insulin [47]. In this study no differences were found in average daily glucose, time spent in the different ranges by GDM and NGT women and maternal and fetal outcomes [47].

In our cohort of false positive women needing insulin, despite having had the CGM inserted on average 27 days after the OGTT (hence around 2 weeks after the GDM education), they had minimal CGM variations and good TIR, but they were then considered in need of insulin because of their pre/post prandial BGL. The role of insulin in determining the presence of GDM is unclear.

In our study GDM women were more often below and above the range, although 42% of NGT participants were found below and 30% above the range during the day and 10% of them had less than 70% of time in range during the night. This was also observed in a study where CGM revealed hyperglycaemia among 16 supposedly NGT obese pregnant women previously tested negative by OGTT [48]. CGM can potentially reduce false-negative OGTT through its more comprehensive glucose profiling.

#### 3.7.10. Machine Learning and CGM

We applied machine learning techniques for the first time to assess the ability of CGM to predict OGTT results based on dual and multiple parameters. In our analysis, a combination of SD and MAGE appeared most predictive: both being elevated in pregnant women with impaired glucose tolerance [49]. Multiple parameter analysis showed RUSBoosted Trees based on CGM sensor mean, SD of the mean and MAGE to have an accuracy percentage of 80% compared to OGTT. Since we are relying on OGTT results and using them as our ground truth to train and test machine learning models, we propose that our high percentage of false classifications (i.e., false positive = 18% and false negative = 23%) may well be due to machine learning models picking up complementary information; therefore, such models could potentially help advance the state of the art to improve OGTT results. We also demonstrated with machine learning that a combination of CGM patterns and risk factor data is potentially able to identify both false positives and negatives from the OGTT. Machine learning has previously been applied only to predict and not qualify the OGTT results [50].

To our knowledge, this is the first study to combine both demographic and CGM risk factors to evaluate accuracy of OGTT diagnosis of GDM. Despite low numbers and the relatively low variability in demographic risk factor scores, a high rate of false-positive diagnosis from OGTT was suggested. Forty-three percent of women having a CDRFS above the mean value of 2.16 had a negative OGTT, of whom 17% had CGMSV above the cut-off linked to GDM. Conversely, 52% of women with a CDRFS below the mean value had a positive OGTT result, of which only 54% showed high variability at CGM, suggesting a high false-positive diagnosis rate from OGTT. Previous research has shown that a significant proportion of women evaluated tested positive with OGTT, despite not showing significantly higher home glucose levels than controls [46].

#### 3.7.11. Strengths, Limitations and Future Directions

The strengths of this study include the relatively large size for a pilot study, the correlation with preliminary findings on CGM, the use of a prediction model applying neural network analysis and the triangulation analysis between OGTT results, CGM parameters, and risk factors. The triangulation analysis suggests potential identification of false negative and positive OGTT diagnoses. However, the shortfalls of OGTT represent a significant hurdle to any study assessing a new diagnostic tool for GDM including CGM. We have recently published a systematic review looking at the numerous alternate markers for establishment of a diagnosis of GDM [51]. This will guide our future work. Future studies need to explore in sufficient numbers the pregnancy outcomes and measures representing ‘true' diagnosis of GDM to fully evaluate OGTT misdiagnoses.

The sample size of this study allowed only for the conceptualization of triangulation and machine learning application to CGM to assess it as a diagnostic test for GDM. GDM women participated up to a month from their GDM education session, when some dietary and exercising habits might have already been modified, although before meeting with the dietitian to start a tailored diet. This may have influenced the number of potential false positives. CGM data following the use of acetaminophen (paracetamol), considered to alter the accuracy of CGM [52], was not excluded due to the low reliability and completion rate of the paper diaries for diet/trainings and medications. It should however be noted that this is likely to have minimal effect on our findings due to general reluctance of women to take any medication in pregnancy [53], and also that acetaminophen effect is thought to be false elevation of CGM readings, whereas our findings were of potential false positives of OGTT, the opposite direction to expected if CGM readings were falsely elevated. To maximize CGM acceptability as a diagnostic test for GDM, women were requested to calibrate the CGM sensor via finger pricks at least twice a day, minimal requirement specified in the Medtronic iPro2 manual, and not four times a day as optimally recommended [54]. Only a quarter of our sample of women (26%) coped with four finger prick calibrations a day; they had all already tested positive for GDM, and following education, were more committed to tightly monitor their glycaemic levels. To overcome the potential loss of data generated for women with less than 4 calibrations per day, CGM days with less than 288 measurements a day were not analyzed as described in our methods [43]. Our results show that there was not significant variation of the delta between the glucose values measured with SBGM versus CGM if women were doing more or less than four calibrations a day, demonstrating good reliability of CGM measurements alone. There was no significant difference in percentage of women with hypoglycaemia in the first versus subsequent nights of CGM monitoring. The mean glucose value measured from the sensor was significantly lower in night one and two compared to night three when the analyzed period included the night following insertion. This is in line with the results of the study by Singh, who did not include night 1 of CGM data in the analysis having demonstrated lower values measured by CGM during the first night of wearing as opposed to the second and the third [43]. Nonetheless, our results demonstrate that the total accuracy and the accuracy of the second morning of wearing, in terms of delta between SBGM and CGM measurements, was not impacted by the lower measurements of night 1 when the first night of CGM monitoring after insertion was considered. To explain the lower values of the first night of monitoring, it should be considered that in our study most of the women (86%) for which the first night of CGM monitoring was included had already been diagnosed with GDM. This might have influenced their diet leading to better glucose control during the SBGM and CGM monitoring period and reduced events of hypoglycaemia in the following nights. In the study by Singh, instead, women had the sensor inserted on the night of their early OGTT (8-19+ 6 weeks): factor that might have influenced the erratic trends of glucose during the first night and influenced the “prolonged segments of low glucose values (2.2 mmol/L, 40 mg/dl)” reported. In the only patient of our cohort with most readings at 2.2 during the first night of monitoring, the delta on the following morning was still lower than in the other days confirming unaltered accuracy. For these reasons, to allow a comprehensive analysis of CGM data over the observed 72 hours period, we still considered valid the CGM data on the night of the insertion and included the full 3 days and 3 nights of CGM data.

Assessing seven full days of CGM data (versus 3 consecutive, sufficiently complete days of the seven days worn), with standardized time frame for the whole cohort, could offer a more complete picture of CGM variability and time in ranges.

The possibility to wear new generation CGM devices on areas different from the pregnant abdomen will most likely increase patients' compliance, while offering more accurate readings.

The retrospective analysis of data created from participants for this study exposes it to data incompleteness. Data on meals, trainings, and drugs were scarce and so not analyzed. The option to record meals, trainings, and drugs digitally could certainly increase the participants' compliance. Further research is needed to confirm and strengthen the results of this study with a larger scale, multicenter study.

## 4. Conclusions

We have found CGM to be feasible, acceptable, and well-tolerated with few adverse effects within a mixed population of pregnant women labelled as NGT and GDM. CGM provided insights into individual glucose variability differences between the two groups and may have identified suspected false positive and negative diagnoses of OGTT. CGM is unique in its capacity to assess diurnal patterns of glucose metabolism under ambulatory conditions, enabling evaluation of glucose metabolism in response to normal individual diet (not the supraphysiological sugar load of the OGTT). CGM has the potential to individualize screening for GDM. Future evaluation of participants stratified into groups according to GDM risk factors may further clarify the utility of CGM in this area. Further, appropriately powered research is required to assess the relationship between CGM cut-off values and GDM risk factors and related complications, such as macrosomia and induced labor, as well as upcoming biomarkers described in the literature, to investigate the validity of CGM as a diagnostic tool compared to, and independent of, the OGTT.

CGM could potentially revolutionize GDM diagnosis and management. We demonstrated this method to be acceptable to patients, correlating well with and potentially unmasking false diagnosis of the current diagnostic method for GDM, the OGTT.

## Figures and Tables

**Table 1:** Continuous glucose monitor parameters used for data analysis.

Sigle - name	Definition/cut-off
Mean	Mean of blood glucose level registered at CGM [24]
SD–Standard deviation	Dispersion of the dataset relative to its mean [24]
CV–Coefficient variation	Mean corrected for SD (SD/mean) [25]
TIR–Time in range	3.5-7.8 mmol/L [16]
TBR–Time below range	=3.0–3.4 mmol/L, 2 = <3.0 mmol/L [16]
TAR–Time above range	>7.8 mmol/L [16], 2= > 10 mmol/L
MAGE–Mean amplitude of glycaemic excursion	Measure of intra-day glycaemic variability [24]
MODD–Mean of daily differences	Measure of inter-daily glycaemic variability [24]

**Table 2:** Demographic statistics of participants.

	GDM (*n* = 40) *n* (%)	NGT (*n* = 33) *n* (%)	*p* value
		
High risk background^∗^	13 (37)	6 (19)	0.08
Primigravida	16 (42)	21 (78)	**<0.01**
Previous GDM	3 (7)	4 (13)	0.69
Family history of DM	23 (57)	7 (23)	**<0.01**
	**Mean ± SD**	**Mean ± SD**	
Age	32.7 ± 5.9	33.8 ± 4.2	0.18
BMI	25.01 ± 6.7	24.34 ± 4.6	0.12
OGTT			
-fasting	5.0 ± 1.7	4.4 ± 0.4	**0.04**
-1 hour	9.6 ± 2.0	6.9 ± 1.6	**<0.01**
-2 hours	7.9 ± 1.8	5.6 ± 1.0	**<0.01**

^∗^High risk background = Aboriginal and Torres Strait Islander, Melanesian, Polynesian, Chinese, Southeast Asian, Middle Eastern, or Indian background.

**Table 3:** Comparison of glycaemic variability at CGM of GDM and NGT groups.

	GDM (*n* = 40) Mean ± SD	NGT (*n* = 33) Mean ± SD	*p* value
Sensor mean	5.53 ± 0.9	5.06 ± 0.8	**0.03**
Standard deviation	0.99 ± 0.3	0.74 ± 0.2	**<0.01**
Coefficient variation	0.21 ± 0.2	0.15 ± 0.5	0.06
Maximum value	8.49 ± 1.5	7.50 ± 1.2	**<0.01**
MODD	0.96 ± 0.3	0.75 ± 0.2	**<0.01**
MAGE	2.33 ± 0.8	1.73 ± 0.7	**<0.01**

MODD: Mean of daily differences, MAGE: Mean amplitude of glycaemic excursions.

**Table 4:** Comparison of the number of patients with values below and above the range and with 70% of time in range over day and night among the total of the GDM and NGT groups.

	DAY					NIGHT				
	TBR	TBR 2	TAR	TAR 2	>70% TIR	TBR	TBR 2	TAR	TAR 2	>70% TIR
GDM (*n* = 40)	17 (42%)	9 (22%)	**27 (67%)**	4 (10%)	**29 (72%)**	17 (42%)	12 (30%)	7 (17%)	1 (2%)	34 (85%)
NGT (*n* = 33)	14 (42%)	6 (18%)	**10 (30%)**	0	**33 (100%)**	9 (27%)	5 (15%)	1 (3%)	0	30 (90%)
*P*	0.592	0.438	**0.002**	0.084	**0.001**	0.134	0.111	0.051	0.548	0.346

TBR = time below range (<3.5 mmol/L), TBR 2 = <3 mmol/L, TAR = time above range (>7.8 mmol/L), TAR 2= >10 mmol/L, TIR = time in range (3.5-7.8 mmol/L).Note: a single value in each range was sufficient to classify a woman in each category.

## Data Availability

The data that support the findings of this study are not publicly available due to their containing information that could compromise the privacy of research participants but are available from the corresponding author in a de-identified manner upon reasonable request.

## Supplementary Materials

Supplementary Materials (1) Patient feedback questionnaire on CGM acceptability (.docx). On completion of the study, whether termination occurred prematurely or after the allocated 5-6 days, the patients were asked to answer the following questions, circling the number to show whether they more strongly agree with the statement on the left or the right. (2) Data extracted from Obstetrix (.docx). (3) Comparison of the mean percentage of time spent above, below, and in range (expressed in decimals) between GDM and NGT women (.docx). (4**)** Machine learning-based analysis to classify OGTT positive and negative participants (.docx). Using ‘group' as response variable (i.e., OGTT based ground truth), all other variables (predictors) were selected in groups to train and cross validate (5-fold) all models available in Matlab's Classification Learner App. (5) MATLAB code used to generate demographic risk factors scores and a CDRFS based on the correlation between single risk factors and GDM in literature (.docx). (6) Linear regression between each risk score and GDM (.docx). (7) Description of parameters included in the Combined Demographic Risk Factor Score and in each of the CGM Score of Variability with their cut-off values (.docx). (8) Correlation of OGTT and CGMSV1-4 (.docx). (9) Triangulation of the results with the 4 different CGMSV. (.docx). (10) STROBE checklist for observational cohort study (.docx).
