# Supplementary material for: Continuous Glucose Monitoring for the Diagnosis of Gestational Diabetes Mellitus: A Pilot Study
Source: J Diabetes Res. 2022 Oct 17;2022:5142918. doi: 10.1155/2022/5142918 (PMC9592228; doi:10.1155/2022/5142918)
Supplement: Supplementary Materials — (1) Patient feedback questionnaire on CGM acceptability (.docx). On completion of the study, whether termination occurred prematurely or after the allocated 5-6 days, the patients were asked to answer the following questions, circling the number to show whether they more strongly agree with the statement on the left or the right. (2) Data extracted from Obstetrix (.docx). (3) Comparison of the mean percentage of time spent above, below, and in range (expressed in decimals) between GDM and NGT women (.docx). (4) Machine learning-based analysis to classify OGTT positive and negative participants (.docx). Using ‘group' as response variable (i.e., OGTT based ground truth), all other variables (predictors) were selected in groups to train and cross validate (5-fold) all models available in Matlab's Classification Learner App. (5) MATLAB code used to generate demographic risk factors scores and a CDRFS based on the correlation between single risk factors and GDM in literature (.docx). (6) Linear regression between each risk score and GDM (.docx). (7) Description of parameters included in the Combined Demographic Risk Factor Score and in each of the CGM Score of Variability with their cut-off values (.docx). (8) Correlation of OGTT and CGMSV1-4 (.docx). (9) Triangulation of the results with the 4 different CGMSV. (.docx). (10) STROBE checklist for observational cohort study (.docx). [file 5142918.f1.zip › Supplementary material 5.CGM for the diagnosis of GDM (2).docx]

**Supplementary material 5. MATLAB code used to generate demographic risk factors scores and a CDRFS based on the correlation between single risk factors and GDM in literature.**

%dRISK

dRISK1 = [];

dRISK2 = [];

dRISK3 = [];

dRISK4 = [];

dRISK_total = [];

%assuming risk is 1.0 otherwise (or baseline risk=1.0)

for i=1:length(group)

    base_risk = 1.0;

    %disp(ethnicity(i))

    ag = age(i);

    %check if person belongs to high risk or low risk category as per the

    %following: For each one-year increase in maternal age from 18 years, GDM risk for the overall population, Asian, and Europid increased by 7.90%, 12.74%, and 6.52%, respectively.

    %apply weight accordingly

    if(ethnicity(i)==1)

        dr1 = base_risk + base_risk * (ag - 18)* 0.1274;

        %we are increasing risk in small proportion from a baseline risk=1

    else

        dr1 = base_risk + base_risk * (ag - 18)* (0.079+0.0652)/2;

    end

    %if person is overweight and older than 35, risk would be 2.45 times higher

    %

    dr2 = base_risk;

    if(bmi(i)>25 && age(i)> 35)

        dr2 = base_risk * 2.45;

    end

    %if family history is = 1 and age > 30, the risk is 3 times higher

    dr3 = base_risk;

    if(famHIst(i) == 1 && age(i)>30)

        dr3 = base_risk * 3.0;

    end

    %if prevGDM == 1, the risk is 5.24 times higher

    dr4 = base_risk;

    if(prevGDM(i) == 1)

        dr4 = base_risk * 5.24;

    end

    dr_total = dr1 + dr2 + dr3 + dr4;

    %update dRISKs for this person

    dRISK1 = [dRISK1; dr1];

    dRISK2 = [dRISK2; dr2];

    dRISK3 = [dRISK3; dr3];

    dRISK4 = [dRISK4; dr4];

    dRISK_total = [dRISK_total; dr_total];
