# Supplementary material for: Continuous Glucose Monitoring for the Diagnosis of Gestational Diabetes Mellitus: A Pilot Study
Source: J Diabetes Res. 2022 Oct 17;2022:5142918. doi: 10.1155/2022/5142918 (PMC9592228; doi:10.1155/2022/5142918)
Supplement: Supplementary Materials — (1) Patient feedback questionnaire on CGM acceptability (.docx). On completion of the study, whether termination occurred prematurely or after the allocated 5-6 days, the patients were asked to answer the following questions, circling the number to show whether they more strongly agree with the statement on the left or the right. (2) Data extracted from Obstetrix (.docx). (3) Comparison of the mean percentage of time spent above, below, and in range (expressed in decimals) between GDM and NGT women (.docx). (4) Machine learning-based analysis to classify OGTT positive and negative participants (.docx). Using ‘group' as response variable (i.e., OGTT based ground truth), all other variables (predictors) were selected in groups to train and cross validate (5-fold) all models available in Matlab's Classification Learner App. (5) MATLAB code used to generate demographic risk factors scores and a CDRFS based on the correlation between single risk factors and GDM in literature (.docx). (6) Linear regression between each risk score and GDM (.docx). (7) Description of parameters included in the Combined Demographic Risk Factor Score and in each of the CGM Score of Variability with their cut-off values (.docx). (8) Correlation of OGTT and CGMSV1-4 (.docx). (9) Triangulation of the results with the 4 different CGMSV. (.docx). (10) STROBE checklist for observational cohort study (.docx). [file 5142918.f1.zip › Supplementary material 9. CGM for the diagnosis of CGM (2).docx]

**Supplemetary material 9. Triangulation of the results with the 4 different CGMSV.**
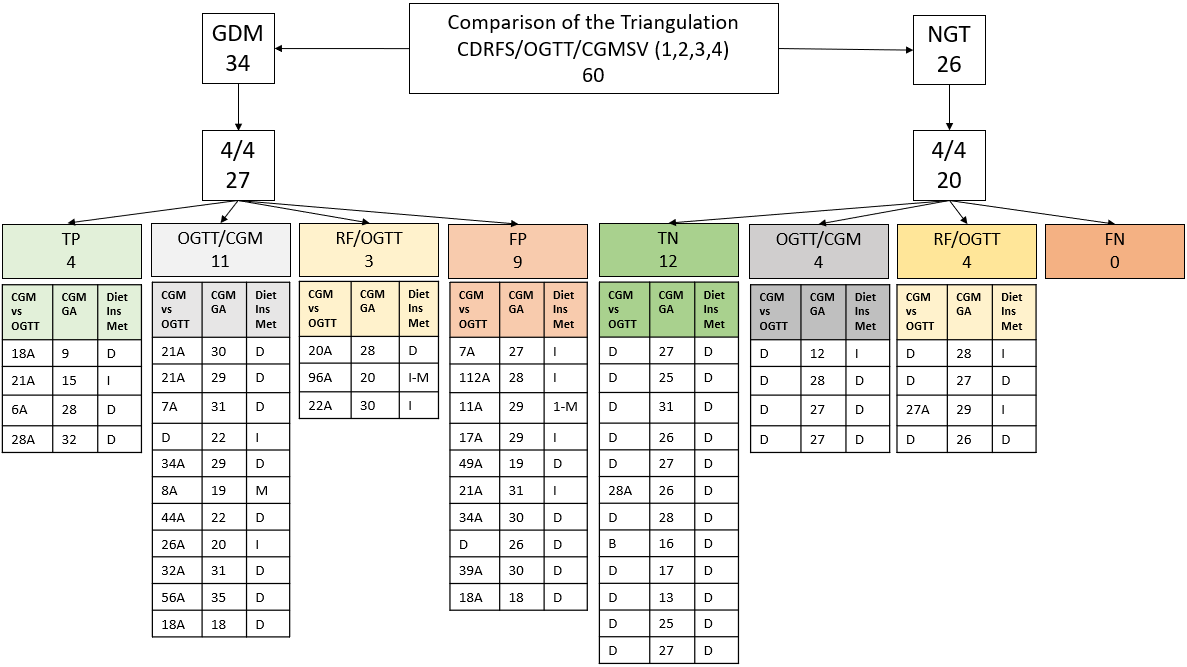


GDM= gestational diabetes mellitus, CDRFS= combined demographic risk factor score, OGTT= oral glucose tolerance test, CGMSV= continuous glucose monitoring score of variability, NGT=normal glucose tolerance test, TP=true positove, OGTT/CGM= risk factors discordant with Ogtt and CGM, RF/OGTT= CGM discordant with RF and OGTT, FP=false positive, TN=true negative, FN=false negative.CGM vs OGTT: timing of CGM wearing vs OGTT: number of days and before (B) or after (A), CGM GA= gestational age in weeks at CGM monitoring, Diet (D) Ins (I) Met (M) = management
